# Platelet-rich plasma combined with lyophilizing thrombin powder for the treatment of complicated enterocutaneous fistula: a case report

**DOI:** 10.3389/fsurg.2023.1252045

**Published:** 2023-10-20

**Authors:** Chao He, Kui Liu, Zhijie Zhao, Zhihao Lai, Linlin Qu

**Affiliations:** Department of Hepatobiliary and Pancreatic Surgery, The Affiliated Hospital of Qingdao University, Qingdao, China

**Keywords:** enterocutaneous fistula, nutrition, platelet-enrich plasma, lyophilizing thrombin powder, seal, case report

## Abstract

**Background:**

Enterocutaneous fistula is one of the most challenging problems facing surgeons. In severe cases, a large amount of fluid loss can lead to problems such as water and electrolyte acid-base imbalance, malnutrition, infection, and organ dysfunction. Here we reported a case of platelet-rich plasma combined with lyophilizing thrombin powder for the treatment of complicated enterocutaneous fistula.

**Case presentation:**

A 48-year-old male, more than 2 years after the operation of abdominal trauma, the leakage of the fistula in the right upper abdominal wall was accompanied by fever for 3 days. The Contrast Fistulography and upper abdomen CT accurately depicted the entry of the meglumine diatrizoate into the small intestine through the small fistula. The patient had a large abdominal wall defect and severe intestinal adhesions. Reoperation may lead to more serious ECF. Therefore, we decided to seal the fistulas with PRP combined with lyophilizing thrombin powder.

**Conclusions:**

The findings in this case report suggest that the combination of PRP and lyophilized thrombin powder holds promise as a viable approach for managing ECF in patients with chronic abdominal wall fistulas, as it appears to facilitate fistula closure, reduce healing time, and improve patient outcomes

## 1. Introduction

Enterocutaneous fistula (ECF) is a pathological channel that develops between the intestinal tract and the skin of the abdominal wall (1). The channel consists of three main parts: the internal fistula opening, fistula tract, and external opening. Likely reasons include secondary pathological reactions after trauma, inflammatory bowel disease, radiation enteritis, severe acute pancreatitis and cancer. Notably, ECF frequently occur in certain congenital diseases. Surgery is the most common cause, accounting for about 75% to 85% (2). After the formation of ECF, with the continuous loss of digestive juice, electrolytes and nutrients are also lacking. At the same time, a large amount of digestive juice overflows, which will also cause erosion or infection of the skin around the fistula. This condition often has a significant negative impact on the patient's quality of life. Therefore, most of the patients require prolonged courses of anti-infective treatment (1). A multimodal approach is necessary for ECFs, as they often occur in patients who are not suitable for surgery and have a high mortality and morbidity rate (3).

In the past, there was insufficient understanding of the pathophysiological process of ECF, and surgical treatment was the main method. ECF are debilitating and often require prolonged preoperative optimization before surgical repair is attempted. In addition, it also has some disadvantages that cannot be ignored, such as large trauma, slow recovery, and high treatment cost. With the continuous development of non-surgical treatment of ECF, the treatment for ECF has gradually changed from simple surgical treatment to non-surgical treatment with the core being comprehensive treatment. The cause of ECF is not only caused by fistula infection and tissue edema, but also related to systemic factors such as poor nutritional status of the patient itself and internal environment disorder. Therefore, the comprehensive treatment based on nutritional support and systemic anti-infection is the main treatment for ECF, and at the same time, non-surgical intervention is performed on the fistula.

PRP is an autologous platelet plasma concentrated through centrifugation, containing high levels of growth factors such as Fibroblast Growth Factors (FGF), platelet-derived growth factor (PDGF), transforming growth factor β (TGF-β), and vascular endothelial growth factor (VEGF). These growth factors promote fibroblast proliferation, enhance extracellular matrix production, and expedite tissue healing and regeneration (4–6). PRP has demonstrated success in various medical fields, including wound healing, bone regeneration, osteoarthritis, and skin conditions (4, 7, 8). Furthermore, PRP contains white blood cells and antibacterial proteins, such as platelet factor 4 (PF-4), connective tissue activating peptide III (CTAP-III), and pro-platelet basic protein (PPBP), which contribute to infection control and reduced inflammation (9).

Given the challenges in managing complicated ECFs and the limitations of available treatments, including the high mortality rate, we propose an innovative approach involving PRP combined with lyophilized thrombin powder to seal ECFs, particularly in cases with chronic wounds.

## 2. Case presentation

A 48-year-old man was admitted to the Department of Hepatobiliary and Pancreatic Surgery on 15 February 2022 with the leakage of the fistula in the right upper abdominal wall was accompanied by fever for 3 days. The patient was admitted to the local hospital after an accident on December 10, 2019. He fell from a height, resulting in duodenal rupture, traumatic injury of bile duct, portal vein rupture with thrombosis, multiple rib fractures, hemoperitoneum, small-bowel edema, and pancreatic head contusion. The patient underwent Repair of portal vein injury, gastrojejunostomy, duodenojejunostomy, jejunum-jejunum anastomosis, and cholecystostomy. On postoperative day 9, the patient presented with ECF. He reached the emergency department of our hospital and underwent an emergency laparotomy, intraperitoneal catheter drain, and jejunostomy. The patient presented with skin ulceration and fistula formation in the right upper abdomen approximately five months ago. There has been a daily discharge of approximately 5 ml of yellow-white purulent fluid. Following wound cleaning, the skin has exhibited intermittent healing. In addition to the aforementioned symptoms, the patient has been experiencing fever and chills, with a body temperature peaking at 39°C. Regrettably, the patient did not seek medical attention promptly upon the onset of these symptoms. The patient has denied any history of allergies, asthma, or exposure to pets. Additionally, there is no known family history of genetic disorders on either the father's or mother's side.

**Figure 1:**
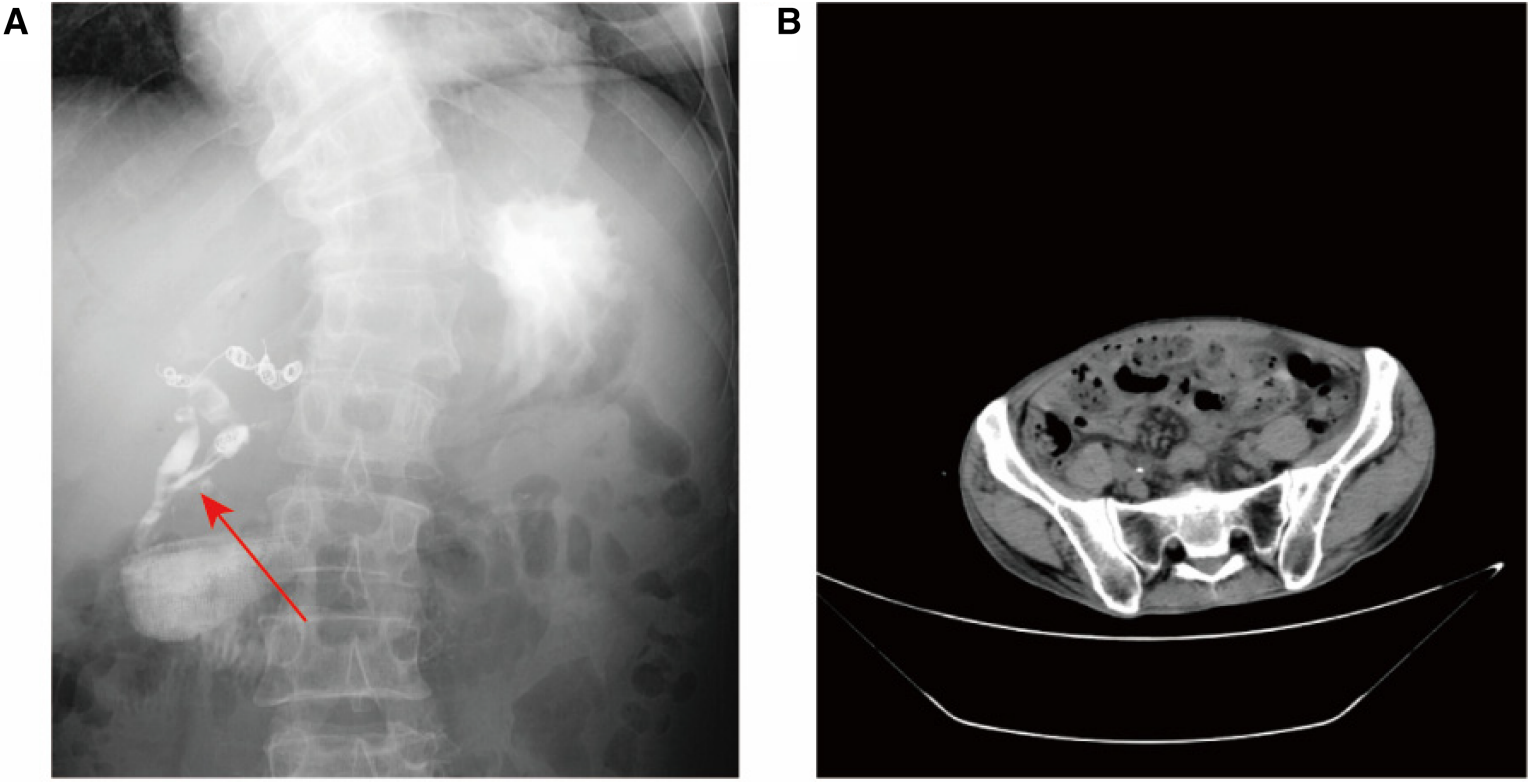
Imaging examination. (**A**) Coronal radiographic image of the fistula or fistula tract before the patient's treatment was blocked. Fistula tract connected to abdominal wall (shown by arrow), scoliosis; (**B**) Abdominal CT suggests ureteral calculus.

The abdominal wall exhibits a subcutaneous tissue defect measuring approximately 10 × 8 cm. Additionally, there is a fistula opening in the right upper abdomen that measures approximately 2 mm in diameter. Yellow-white liquid is observed flowing out from the fistula opening, and the surrounding skin appears corroded and damaged. The abdomen was tender in right upper quadrant and soft, with no rebound tenderness. Lab data revealed white blood cell 16.78 × 10^9^/L, neutrophil count 15.11 × 109/L, neutrophil ratio 90.10%, platelet 55.00 × 109/L, plateletcrit (PCT) 0.06%, C-reactive protein 70.38 mg/L, Carbohydrate Antigen 19–9 (CA19–9) > 1000.00 U/ml, Carbohydrate Antigen 24–2 (CA24–2) 214.70 IU/ml, Total protein (TP) 46.3 g/L, Albumin 25.8 g/L, prealbumin 80 mg/L, total bilirubin (TB) 64.5 umol/L, direct bilirubin (DB) 40.1 umol/L, alanine aminotransferase (ALT) 193.8 U/L, aspartate aminotransferase (AST) 153.4 U/L, γ-glutamyltransferase (GGT) 536.7 U/L, alkaline phosphatase (ALP) 545.2 U/L. No positive findings were obtained from routine blood coagulation and markers of renal function. Contrast Fistulography and upper abdomen CT showed contrast entering the small intestine through the fistula, suggesting a (small bowel) intestinal fistula (Figure 1A).

Based on the patient's symptoms, abdominal examination, and imaging findings, the admission diagnosis was complicated ECF, ureteral stones (Figure 1B), scoliosis. In addition, the patient had a history of gastrointestinal surgery and biliary tract surgery. Due to a significant defect in the patient's abdominal wall, the intestinal canal is situated below the epidermis of the abdominal wall. Additionally, the patient has undergone multiple operations resulting in severe intestinal adhesion. Firstly, this conservative approach involved flushing the fistula with physiological saline while simultaneously administering antibiotics for infection control and providing nutritional support to the patient. Conservative treatment had proven ineffective in managing the patient's condition. Futhermore, reoperation may lead to more serious ECF. Therefore, we decided to seal the fistulas with PRP combined with lyophilizing thrombin powder.

**Figure 2:**
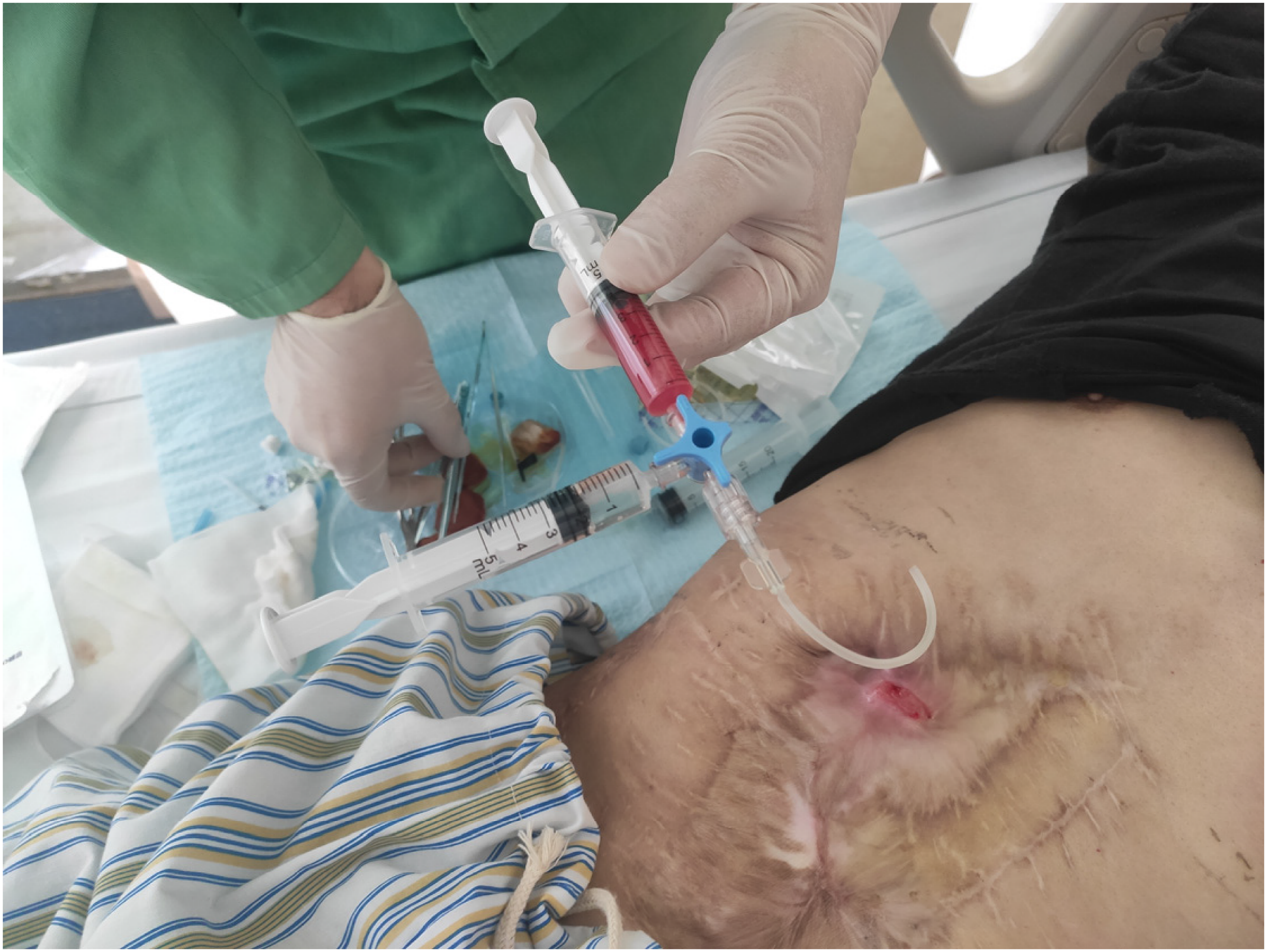
The PRP injection process, with the tube extending from the device to the treatment site as well as the injection of PRP and thrombin into the union tee.

**Figure 3:**
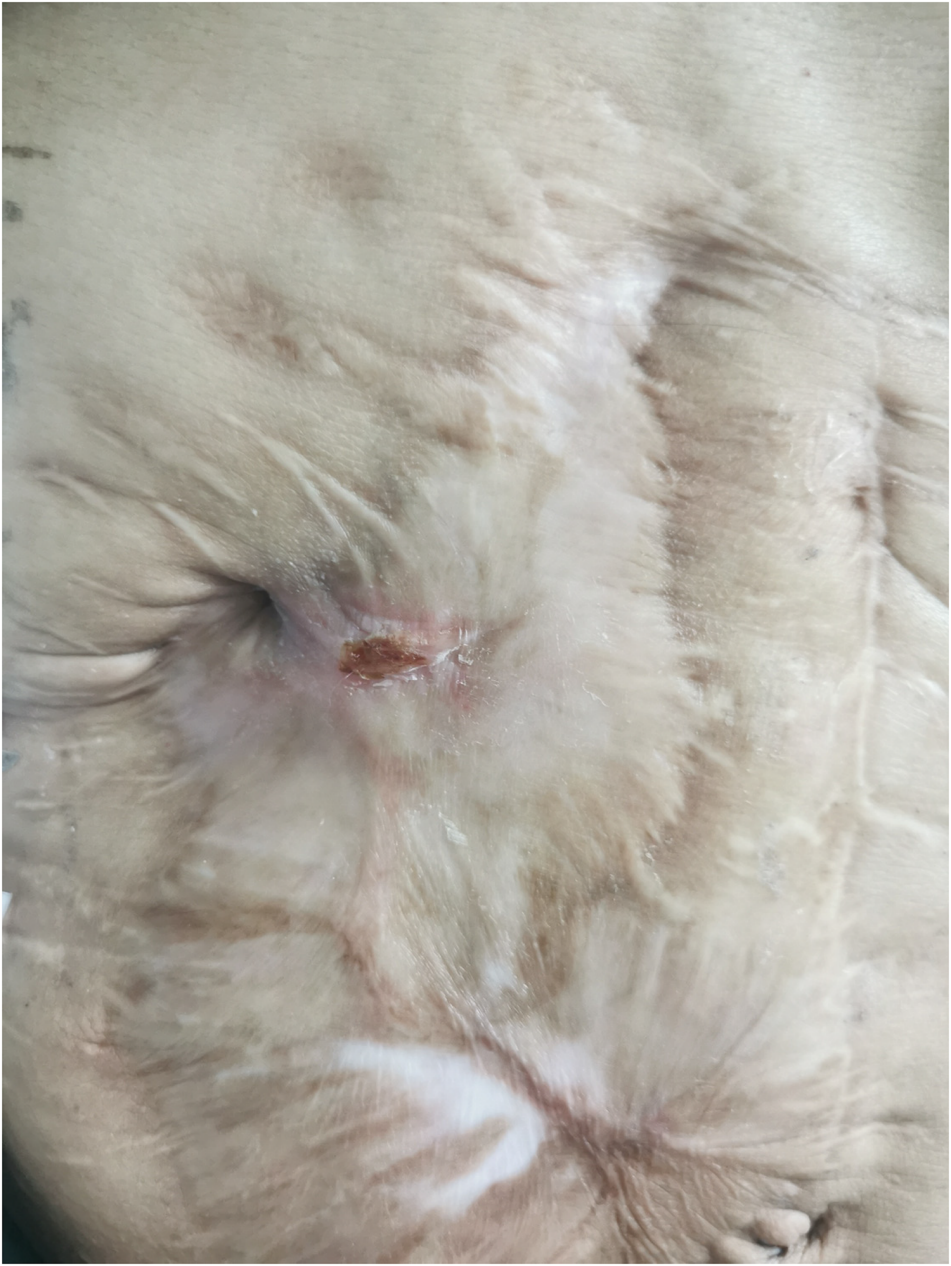
The drain site on day 5 after treatment; the wound is closed.

Following the patient's informed consent, we performed daily sinus flushing with normal saline for a period of three days. Subsequently, we collected 300 ml of the patient's autologous venous blood and separated approximately 15 ml of platelet-rich plasma. To prepare the thrombin solution, 5,000 units of thrombin freeze-dried powder were dissolved in 10 ml of 3% calcium chloride solution. Platelet-rich plasma and the thrombin solution were drawn into separate syringes and connected using a three-way device. The mixture was then injected through the fistula and pressure was applied for 5 min (Figure 2). Following the successful occlusion of the fistula, the patient's body temperature was continuously monitored, registering at 37.1°C. On the second day of treatment, a subsequent blood routine test was conducted, revealing a white blood cell count of 4.57 × 10^9^/L. Importantly, these observations presented no deviations from the expected values, thereby affirming the absence of any discernible inflammatory response. After 5 days, the fistula was sealed and no further leakage of intestinal fluid was observed (Figure 3). At the one-year follow-up, the patient reported the absence of abdominal distension and abdominal pain, with no reported complications.

## 3. Discussion

Complicated ECF often results from abnormal growth factors and inflammation. Traditional therapies like dressings and fibrin glue fall short in providing adequate healing due to the lack of necessary growth factors. To address this, we use PRP combined with Lyophilizing Thrombin Powder instead of conventional fibrin glue. PRP, with its growth factor content, is a suitable and safe treatment for chronic wounds with poor blood supply. Additionally, PRP's leukocytes have anti-inflammatory and antimicrobial effects. The fibrinogen-thrombin complex contains growth factors aiding tissue regeneration and wound healing acceleration.

In cases with stable sinus drainage, we recommend fibrin glue closure. Fibrin glue is user-friendly, safe, and increasingly used clinically (10). Numerous studies have demonstrated the effectiveness of PRP in treating different types of fistulas, yielding encouraging results. For instance, a recent study found that PRP could accelerate fistula closure and shorten healing time in ECF patients without systemic sepsis or peritonitis (11). In another study, PRP injection into a vesicovaginal fistula resulted in a clinical cure for 92% of patients after six months of follow-up (12). Additionally, PRP has been shown to be effective in treating tracheobronchial and anal fistulas in other studies (13, 14). In a pilot study, the combination of mucosal push valve and platelet-rich plasma has moderate efficacy in the treatment of high perianal fistula associated with Crohn's disease, with a cure rate of 70% (15). In a study by Wu et al. (16), involving 145 patients, the use of autologous platelet rich fibrin glue (PRFG) proved to be effective in treating low output fistulas in patients with parenteral fistulas. The study showed that PRFG resulted in shorter fistula closure times and improved closure rates, without any adverse effects. Another study by Mizushima et al. (17), involving a small sample size of six patients, demonstrated that autologous adipose-derived regenerative cells (ADSC) transplanted with fibrin glue were safe and feasible for the closure of extra-intestinal fistulas, with a success rate of 100%. These findings suggest that PRFG and ADSC may be promising treatment options for fistulas.

The variations in clinical research data among different scholars can be attributed to the inconsistent basic conditions of the patients. our study is the first to report on the effectiveness of PRP combined with lyophilizing thrombin powder therapy for treating complicated ECF, and provides evidence on which patients are more likely to benefit from this treatment. As demonstrated in this case, the treatment effectively reduces the patient's pain by facilitating the closure of the fistula and shortening the healing time. It is anticipated that further progress in the treatment of ECF will be made with the advancement of medicine, material science, and other related disciplines.

## 4. Conclusion

In conclusion, the innovative approach of using PRP combined with lyophilized thrombin powder for the treatment of chronic abdominal wall fistulas, particularly in cases of complicated ECF, shows promise as a cost-effective and reliable option for surgeons when clinical consensus or guidelines are lacking, highlighting the potential benefits for improving patient outcomes and reducing healing time.

## Data Availability

The original contributions presented in the study are included in the article/Supplementary Material, further inquiries can be directed to the corresponding author.
